# Detyrosinated microtubule arrays drive myofibrillar malformations in *mdx* muscle fibers

**DOI:** 10.3389/fcell.2023.1209542

**Published:** 2023-08-25

**Authors:** Anicca D. Harriot, Tessa Altair Morris, Camilo Vanegas, Jacob Kallenbach, Kaylie Pinto, Humberto C. Joca, Marie-Jo Moutin, Guoli Shi, Jeanine A. Ursitti, Anna Grosberg, Christopher W. Ward

**Affiliations:** ^1^ Department of Biochemistry and Molecular Biology, University of Maryland School of Medicine, Baltimore, MD, United States; ^2^ Center for Complex Biological Systems, Edwards Lifesciences Foundation Cardiovascular Innovation and Research Center, and the NSF-Simons Center for Multiscale Cell Fate Research, University of California, Irvine, Irvine, CA, United States; ^3^ Department of Orthopedics, University of Maryland School of Medicine, Baltimore, MD, United States; ^4^ Department of Molecular Medicine, University of Maryland School of Medicine, Baltimore, MD, United States; ^5^ INSERM U1216 Centre National de la Recherche Scientifique, Grenoble Institut Neurosciences, University Grenoble Alpes, Grenoble, France; ^6^ Department of Biomedical Engineering, Sue and Bill Gross Stem Cell Research, University of California, Irvine, Irvine, CA, United States; ^7^ Department of Chemical and Biomolecular Engineering, Sue and Bill Gross Stem Cell Research, University of California, Irvine, Irvine, CA, United States

**Keywords:** dystrophy, skeletal muscle, myoarchitecture, microtubule array, detyrosination

## Abstract

Altered myofibrillar structure is a consequence of dystrophic pathology that impairs skeletal muscle contractile function and increases susceptibility to contraction injury. In murine Duchenne muscular dystrophy (*mdx*), myofibrillar alterations are abundant in advanced pathology (>4 months), an age where we formerly established densified microtubule (MT) arrays enriched in detyrosinated (deTyr) tubulin as negative disease modifiers impacting cell mechanics and mechanotransduction. Given the essential role of deTyr-enriched MT arrays in myofibrillar growth, maintenance, and repair, we examined the increased abundance of these arrays as a potential mechanism for these myofibrillar alterations. Here we find an increase in deTyr-tubulin as an early event in dystrophic pathology (4 weeks) with no evidence myofibrillar alterations. At 16 weeks, we show deTyr-enriched MT arrays significantly densified and co-localized to areas of myofibrillar malformation. Profiling the enzyme complexes responsible for deTyr-tubulin, we identify vasohibin 2 (VASH2) and small vasohibin binding protein (SVBP) significantly elevated in the *mdx* muscle at 4 weeks. Using the genetic increase in VASH2/SVBP expression in 4 weeks wild-type mice we find densified deTyr-enriched MT arrays that co-segregate with myofibrillar malformations similar to those in the 16 weeks *mdx*. Given that no changes in sarcomere organization were identified in fibers expressing sfGFP as a control, we conclude that disease-dependent densification of deTyr-enriched MT arrays underscores the altered myofibrillar structure in dystrophic skeletal muscle fibers.

## 1 Introduction

Skeletal muscle fibers exhibit highly ordered myofibrillar structure which is essential for efficient force generation. Myofibrils (1 µm diameter) are composed of individual contractile units (i.e., sarcomeres, ∼2 µm in length) arranged in series to span the length of the muscle fiber (500 µm to a few cm). The number of parallel packed myofibrils within the muscle fiber governs the contractile force. Until recently, myofibrils were thought to be independent units co-registered through protein links between their Z-line sarcomere boundaries. However, new evidence of sarcomeres branching between registered myofibrils (Willingham et al., 2020) has redefined these structures as a continuous myofibrillar matrix that facilitates the highly coordinated, unilateral contraction of the muscle fiber.

In contrast to the registered myofibrillar matrix seen in healthy skeletal muscle, are myopathies such as Duchenne muscular dystrophy (DMD) where myofibrils become misaligned and torturous resulting in misorientation of force vectors and dyssynchronous activation of sarcomeres (Head et al., 1997; Buttgereit et al., 2013; Liu et al., 2013; Ding et al., 2018). These changes result in significant reductions in isometric force and velocity of contraction as well as increased shear stress that predisposes damage at these locations (Schneidereit et al., 2018; Stefanati et al., 2021; Ritter et al., 2022). While misalignment of the sarcomeres is now established as pathognomonic in DMD, the mechanisms that predispose their occurrence are unknown.

The cytoskeleton is a dynamic structural and signaling scaffold of microtubule (MT), actin, and intermediate filaments (IF) that is essential for the intracellular trafficking, maintenance of cellular architecture, and positioning of organelles in all cells. In skeletal muscle fibers, microtubules form a geometric array ordered by their interaction with highly structured sarcomeric and membrane spanning complexes. Microtubules garnered early attention in DMD muscle given the disorganized array structure seen early in disease that became densified with disease progression (Prins et al., 2009; Liu and Ralston, 2014; Oddoux et al., 2019). The discovery of dystrophin as an MT binding partner has begun to resolve the mechanisms responsible for the disorganized MT array structure at the membrane of DMD muscle fibers (Prins et al., 2009) and motivated many groups to determine how these MT alterations impact dystrophic pathology.

Microtubules are hollow tube-like structures formed by the dynamic polymerization of α-β tubulin protein dimers. The structure and function of MTs are regulated by post-translational modifications (PTM) to their tubulin monomers. Detyrosination (deTyr), the reversible enzymatic removal of α-tubulin’s COOH-terminal tyrosine, is one such PTM that promotes the interaction of MTs with binding partners (Peris et al., 2009; Aillaud et al., 2017; Nieuwenhuis et al., 2017; Salomon et al., 2022). In striated muscle, MT arrays enriched in deTyr-tubulin have been shown to play a role in the myofibrillar growth, maintenance, and repair of striated muscle through the patterned recruitment of myosin, actin, mRNAs, and ribosomes for the assembly of sarcomeres (Dhanyasi et al., 2021). In fact, an increased level of deTyr-enriched MTs is an early and critical event in sarcomerogenesis (Gundersen et al., 1989; Chang et al., 2002).

Work by our group has identified that deTyr-enriched MT arrays regulate the stiffness of the muscle fiber cytoskeleton and thus the activation of NADPH Oxidase 2 (Nox2) dependent reactive oxygen species (ROS) and calcium (Ca^2+^) signals by mechanotransduction (Khairallah et al., 2012; Kerr et al., 2015). In the murine model of DMD (i.e., *mdx*) we identified the densification of deTyr-enriched MT arrays as a consequence of disease pathology that increases the passive mechanics of muscle fibers. Together with the increased expression of Nox2 proteins, these changes underscore the excess mechanotransduction elicited Nox2-ROS and Ca^2+^ signals linked to dystrophic pathology (Khairallah et al., 2012; Prosser et al., 2013; Kerr et al., 2015).

Our lab’s previous work on dysregulated MT mechanotransduction in murine DMD (*mdx*) focused on murine models between 3–9 months when pathology is entrenched yet progression is evident (Khairallah et al., 2012; Kerr et al., 2015). Within this timeframe of disease, our group and others have previously identified and profiled the increased occurrence of muscle fibers with gross structural malformations (i.e., splitting, branching) (Head et al., 1997; Head, 2010; Lovering et al., 2011; Buttgereit et al., 2013; Ritter et al., 2022) that increase the susceptibility to contractile damage in DMD. Here we were intrigued by work suggesting that these gross alterations in muscle fiber structure arose from structural changes in the myofibrils (Buttgereit et al., 2013). Informed by our observation that the densification of deTyr-enriched MT arrays often occurred in discrete areas in *mdx* muscle fibers(Khairallah et al., 2012; Kerr et al., 2015), and evidence that microtubules are essential for myofibrillar growth, maintenance, and repair (Pizon et al., 2005; Scholz et al., 2008; Denes et al., 2021; Dhanyasi et al., 2021), we hypothesized a link between the disease-altered MT arrays and the occurrence of myofibrillar malformations in DMD.

In the present study, we focused early in disease pathology to bias our capture of the mechanisms that underlie the development of myofibrillar malformations. In muscle fibers from young mice (4 weeks) we identify a significant increase in deTyr-MTs in *mdx,* yet no evidence of myofibrillar malformation above that seen in WT. Profiling muscle fibers at 16 weeks, we find the level of deTyr-tubulin increases disproportionally in the *mdx* where it occurs largely in MT arrays that co-localize with areas of myofibrillar malformation.

Profiling the enzyme complexes responsible for deTyr-tubulin we found vasohibin 2 (VASH2) and SVBP significantly elevated in the *mdx* muscle at 4 and 16 weeks. To determine the consequences of elevated VASH2 activity on myofibrillar structure we overexpressed VASH2 and SVBP in muscles of 4 weeks wild-type mice. Using this gain-of-function approach we show that VASH2/SVBP overexpression modeled the densification of deTyr-MTs seen in the 16 weeks *mdx*. Furthermore, we demonstrate that deTyr-enriched MT arrays co-segregate with myofibrillar malformations comparable to those found in 16 weeks *mdx*. We conclude that disease-altered microtubules are an early event in dystrophic pathology that predisposes the altered myofibrillar structure in dystrophic skeletal muscle fibers.

## 2 Materials and methods

### 2.1 Animal use

All animal protocols were reviewed and approved by the Institutional Animal Care and Use Committee at the University of Maryland (IACUC) and adhere to NIH guidelines. All mice were obtained from Jackson Laboratories (Bar Harbor, MA) and include wild-type (C57BL.10/J, strain #000665) and *mdx* (C57BL.10/J *mdx*, strain #001801) mice at 4 and 16 weeks of age.

#### 2.1.1 Electroporation

Anesthetized mice (2% isoflurane) were injected with 25 µL of 1 mg/mL of hyaluronidase (Sigma-Aldrich) subcutaneously into the sterilized food pad of both hindlimbs. After 1 h, one footpad was injected with plasmid cDNA (20 μL at 1 μg/μL) containing a bicistronic construct of FLAG-VASH2-sfGFP-His (IRES SVBP-myc) (Aillaud et al., 2017) with the contralateral footpad receiving a plasmid cDNA containing FLAG-sfGFP-His as a control (1 μg/μL). The plasmid cDNA was then delivered to the flexor digitorum brevis (FDB) muscle by electroporation through sterile electrodes placed subcutaneously at the proximal and distal ends of the FDB. The pulse protocol consisted of 30 pulses of 150 V for 20 ms duration at a frequency of 1 Hz. Mice were humanely euthanized and FDBs were harvested after 5 days.

#### 2.1.2 Muscle fiber preparation

FDB muscles were harvested bilaterally in sterile mouse ringer and maintained overnight in DMEM (supplier) supplemented with collagenase A (Roche, 0.2 mg/mL) and 1% Penicillin-Streptomycin in a CO_2_ incubator (37°C, 5% CO_2_). Following gentle trituration to yield single FDB fibers, the cells were washed once in DMEM supplemented with 10% fetal bovine serum then washed twice in physiological Ringer solution with 1 mM EGTA (pH, 7.4). Fibers were then either maintained in Ringer solution at room temperature for live cell imaging or fixed in 4% PFA with 5 mM EGTA for 20 min at room temperature, washed twice in PBS then stored in PBS with 0.4% sodium azide until used.

#### 2.1.3 *In vivo* contractile function

Contractile performance and injury susceptibility were tested *in vivo* as described previously (Khairallah et al., 2012; Kerr et al., 2015). Anesthetized mice (2%–3% isoflurane) were placed in a supine position on the temperature-maintained (Deltaphase Isothermal Pad, Braintree Scientific) platform of an Aurora 3100 with the knee stabilized and foot affixed on the footplate of the torque transducer. The plantar flexor muscle group (gastrocnemius, soleus) was activated by percutaneous stimulation. The force frequency relationship was evaluated with 500 msec trains of square pulses (0.1 ms) between 1 and 150 Hz. The susceptibility to contraction force-loss was evaluated with 25 eccentric (i.e., lengthening) contractions.

#### 2.1.4 Mechanical properties

FDBs maintained in Ringer were placed on a glass-bottom dish coated with ECM (E6909; Sigma-Aldrich). The near membrane mechanical properties of the FDB were quantified with a Chiaro nano-indenter (Optics11) using a cantilever (0.044 N/m stiffness) with a round probe (3-µm radius). Indentation (1uM) profiles at speeds from 0.5 to 25 μm/s were analyzed with a Hertzian contact model to calculate the Young’s modulus (i.e., stiffness) of the FDB.

### 2.2 Western blotting

Homogenized cell lysates were processed via SDS-PAGE (Mini-PROTEAN TGX precast gels), transferred to a membrane (Millipore Immobilon-FL PVDF), stained (Revert 700 Total Protein Stain) for 3–5 min at room temperature, then washed in Wash solution (P/N 926-11012). After decanting solution, the membrane was rinsed in ultrapure water and imaged on the LICOR Odyssey CL-x system. Immediately after imaging the membrane was incubated with Revert destaining solution (P/N 926-11013) for 5 min then rinsed in ultrapure water before blocking (SuperBlock PBS 37515; Thermo Fisher Scientific) for 1 h at room temperature. The membrane was probed overnight for β-tubulin (T4026, Sigma-Aldrich), deTyr-tubulin (31-1335-00, RevMAb Biosciences), acetylated tubulin (T7451, clone 6-11B-1; Sigma-Aldrich), and gp91phox (Abcam; ab129068). Blots were washed 3 times for 5 min with 1x TBS + 0.1% Tween20. Blots were then incubated with appropriate corresponding secondary antibody (1:5000) for 1 h at room temperature, washed 3x for 5 min with 1x TBS + 0.1% tween 20 and imaged the LICOR Odyssey CL-x system.

### 2.3 RT-qPCR

Gastrocnemius muscles were collected from 5 *mdx* and 5 wild-type mice at both 4 and 16 weeks of age, snap-frozen in isopentane cooled on dry ice and stored at −80°C. Tissues were later powdered and homogenized in TRI-reagent (Zymo Research). Phase separation was performed using 0.2 mL of chloroform per 1 mL of TRI-reagent, with samples shaken vigorously for 2min then centrifuged at 12,000 × *g* for 10 min at 4°C. To precipitate RNA from the aqueous phase, 0.5 mL isopropyl alcohol per 1 mL TRI-reagent used for lysis was added and incubated at room temperature for 10 min before centrifuging for 10 min at the aforementioned settings. The resulting RNA pellet was washed with 75% ethanol, centrifuged for 5 min at 7,500 × *g* at 4°C, then dissolved in 30 µL DNase-RNase free water at which point RNA concentration was measured using a spectrophotometer. 2.5 µg samples of RNA were reverse transcribed to cDNA using the SuperScript IV First-Strand Synthesis System (Invitrogen), following the manufacturer protocol. cDNA was diluted 1:10 in RNase-free water before use for RT-qPCR. Primers used in this study are listed in Supplementary Table S1 and include primers for 3 housekeeping genes. All reactions were performed using Thermo Fisher Maxima SYBR Green/ROX qPCR Master Mix (2X) on the Applied Biosystems QuantStudio 3 machine with QuantStudio software for cycling and analysis. The DDCt Method was performed against WT values at each age to determine relative gene expression and fold-change.

### 2.4 Immunofluorescence and automated imaging

Fixed FDB fibers were blocked for 2 h at room temperature in Superblock™ Blocking Buffer in PBS (Thermo Scientific) with 0.04% saponin. Fibers were incubated in an Eppendorf tube with primary antibodies to detect microtubule structure (beta-tubulin; T4026, Sigma-Aldrich) and the population of microtubule tubulin modified by detyrosination (deTyr-tubulin; 31-1335-00, clone RM444, RevMAb Biosciences United States, Inc.). To visualize myofibrillar structure, sarcomeric actin was decorated with phalloidin conjugated to Alexa Fluor 633 (A22284, Invitrogen). Primary Antibodies and phalloidin were used overnight at 4°C. The following day FDB fibers were incubated with the appropriate secondary antibodies (diluted in PBS containing 0.04% saponin and 0.1% sodium azide) for 2 h at room temperature, washed three times in PBS, then mounted onto slides with ProLong Gold + Dapi mountant (Invitrogen).

Fixed FDB fibers were imaged on an inverted Nikon C2+ confocal fluorescence system using an automated protocol developed in NIS Elements AR JOBS. Single fibers were identified by their actin labeling (i.e., phalloidin 633) from a full-slide tile scanned image (10x air obj.). Fibers without evidence of bends or hypercontraction under visual inspection (30–50 fibers per slide) were logged as regions-of-interest (ROI). Each identified fiber ROI was imaged using an automated routine that identified the muscle fiber surface and collected a full thickness z-stack (0.5 µm steps; 4 frame average) at 40x (1.4 N.A. Plan Apo air obj.) and 1.3 Airy units which yielded 0.31 mm/pixel resolution.

#### 2.4.1 Automated microtubule structural analysis

The properties of the microtubule network were quantified in NIS Elements AR General Analysis 3. Briefly, an inverse binary mask of the phalloidin label (Cy5 channel) identified areas of myofibrillar structure (black) and areas of myofibrillar gaps (white) linked to the regions of altered continuity. Within each z-stack image, the density of deTyr-tubulin (binarized deTyr-tubulin) was determined within the areas of myofibrillar structure and continuity gaps and normalized to the measured area. The area around the nuclei was masked to exclude any microtubule alterations around the nuclei as a confounding factor. For global density measures, the total deTyr-tubulin stain for each z-slice was normalized to the fiber area (as determined by the phalloidin label).

### 2.5 Statistical methods

Statistical comparisons were with GraphPad Prism v9.3.1. Two group comparisons were with *t*-test and multiple groups comparisons were with ANOVA. The data is presented as Mean ± SEM.

## 3 Results

### 3.1 Young *mdx* mice exhibit functional deficits and microtubule alterations

Our lab’s previous work on dysregulated MT mechanotransduction and gross structural alterations was in *mdx* mice at 3–9 months of age when pathology is well-established and still progressing. In this study, we examined wild-type (C57BL.10/J) and *mdx* (C57BL.10/J *mdx*) mice at 4 and 16 weeks of age to elucidate the mechanisms that underlie the development of myofibrillar malformations.

**FIGURE 1:**
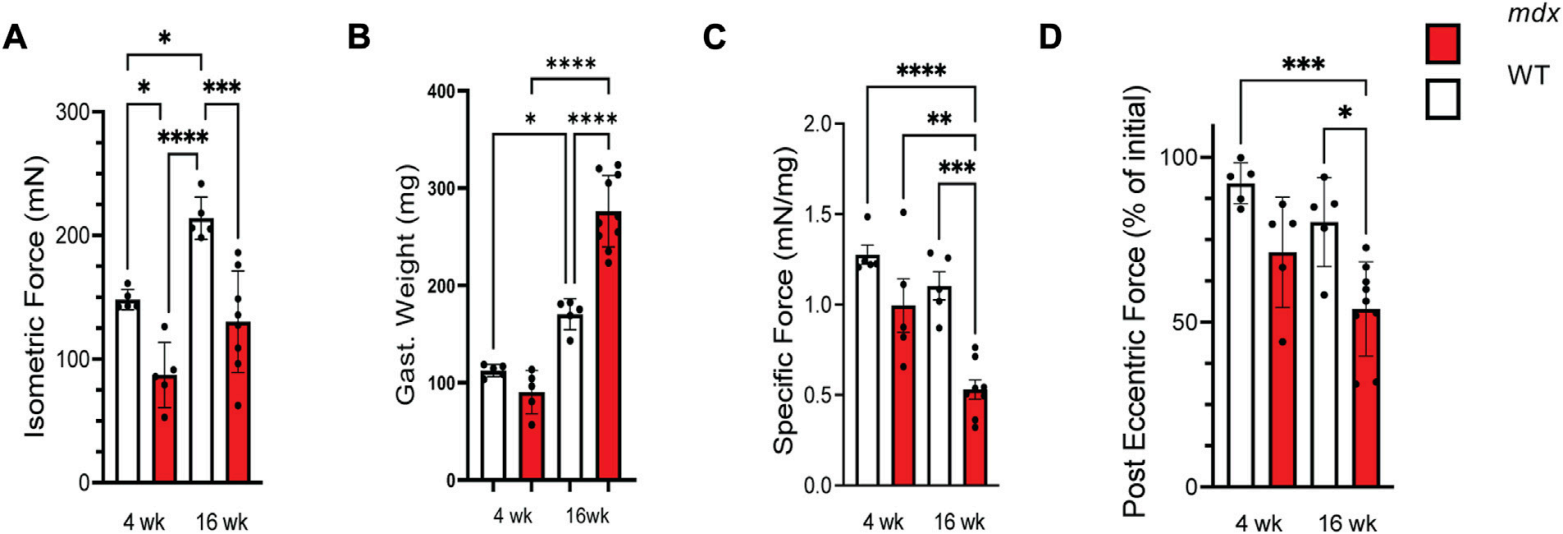
Force production decreases with age in *mdx* while injury susceptibility increases. **(A)** Isometric force produced at 150 Hz in wild-type (white) and *mdx* (red) gastrocnemius muscles at 4 and 16 weeks, respectively. **(B)** Weights of the gastrocnemius muscles from WT and *mdx* mice used to normalize force production for determining **(C)** specific force production in age-matched WT and *mdx* mice. **(D)** Decrement of isometric force in gastrocnemius muscles from 4 to 16 weeks WT and *mdx* mice after 25 eccentric contractions. *Mdx* mice experience increased force loss when compared to wild-type. Values are means ± SEM. All analysis are one-way ANOVA with Šídák’s multiple comparisons test (*p < 0.05; **p < 0.01; ***p <0.001; ****p <0.0001).

Our initial experiments sought to establish the functional status of the muscle at these ages. Evaluating *in vivo* plantar flexor function, we confirmed deficits in maximal isometric force in the *mdx* at both 4 and 16 weeks (Figure 1A). Measuring the weight of the gastrocnemius muscle we identified no differences between genotypes at 4 weeks yet a significant increase in the mass of the *mdx* gastrocnemius at 16 weeks, a finding consistent with the pseudohypertrophy reported at this age (Figure 1B). Calculating the specific force (i.e., force normalized to mass) revealed no difference between genotypes at 4 weeks yet a significant drop in the specific force of the *mdx* was observed at 16 weeks (Figure 1C). Finally, evaluating isometric force loss following 20 *in vivo* eccentric contractions we again found no significant deficit between genotypes at 4 weeks; this however, progressed to a significant decrease in *mdx* at 16 weeks (Figure 1D). Taken together, we identified an acceleration in functional deficits after 4 weeks of age in the *mdx*.

**FIGURE 2:**
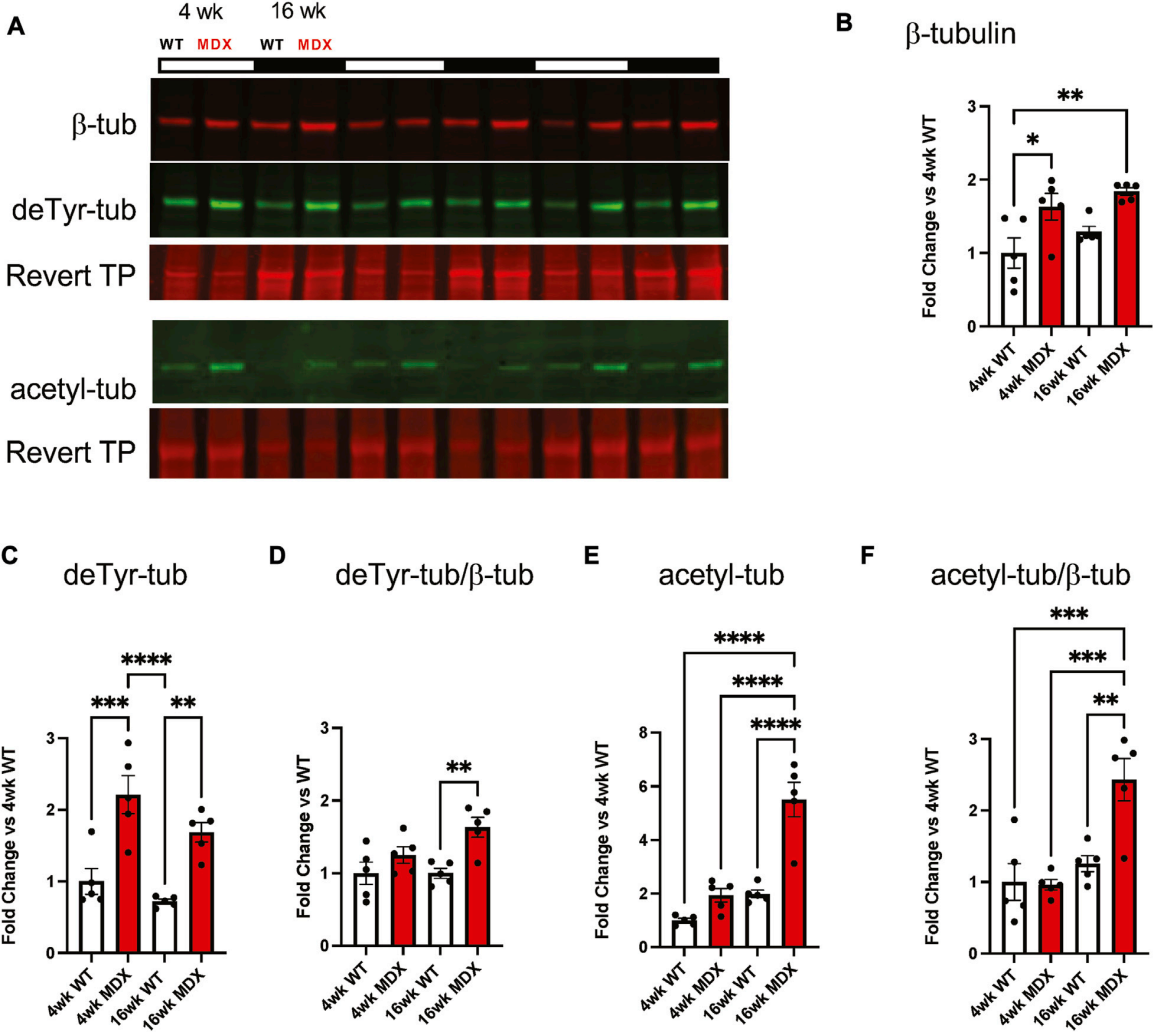
Detyrosination increases with dystrophic disease progression. **(A)** Western blots of the gastrocnemius muscle from wild-type and *mdx* mice at 4 weeks (*n* = 5) and 16 weeks (*n* = 5) demonstrate **(B)** increased tubulin abundance and post-translational modification by **(C)** detyrosination.**(D)** which is a disproportionate increase when compared to tubulin abundance. **(E)** We see a significant increase in acetylation which is **(F)** further confirmed as disproportionate when compared to β-tubulin. Values are means ± SEM. All analysis are one-way ANOVA with Šídák’s multiple comparisons test (*p < 0.05; **p < 0.01).

Our group, and others (Iyer et al., 2017; Loehr et al., 2018; Nelson et al., 2018; Nelson et al., 2020), have implicated the proliferation MT arrays as negative disease modifiers in adult *mdx* mice with advanced pathology. Western blot profiling of gastrocnemius muscle from 4 week old *mdx* vs. WT mice identified a significant increase in tubulin expression (Figure 2B) and its modification by detyrosination (deTyr-tub; Figure 2C), yet we found tubulin acetylation unchanged (acetyl-tub; Figure 2E). Profiling 16 weeks WT muscle we found tubulin expression and levels of deTyr-tub and acetyl-tub unchanged vs. their 4 weeks counterparts. Profiling the 16 weeks *mdx* we found a similar elevation in tubulin expression (Figure 2B) and level of deTyr-tub as seen at 4 weeks with now a significant elevation in acetyl-tub. Normalizing the level of tubulins modification to its expression we find both deTyr-tub and acetyl-tub increase disproportionately to tubulin expression at 16 weeks (Figures 2D, F).

### 3.2 *mdx* mice exhibit malformed myofibrillar structure

**FIGURE 3:**
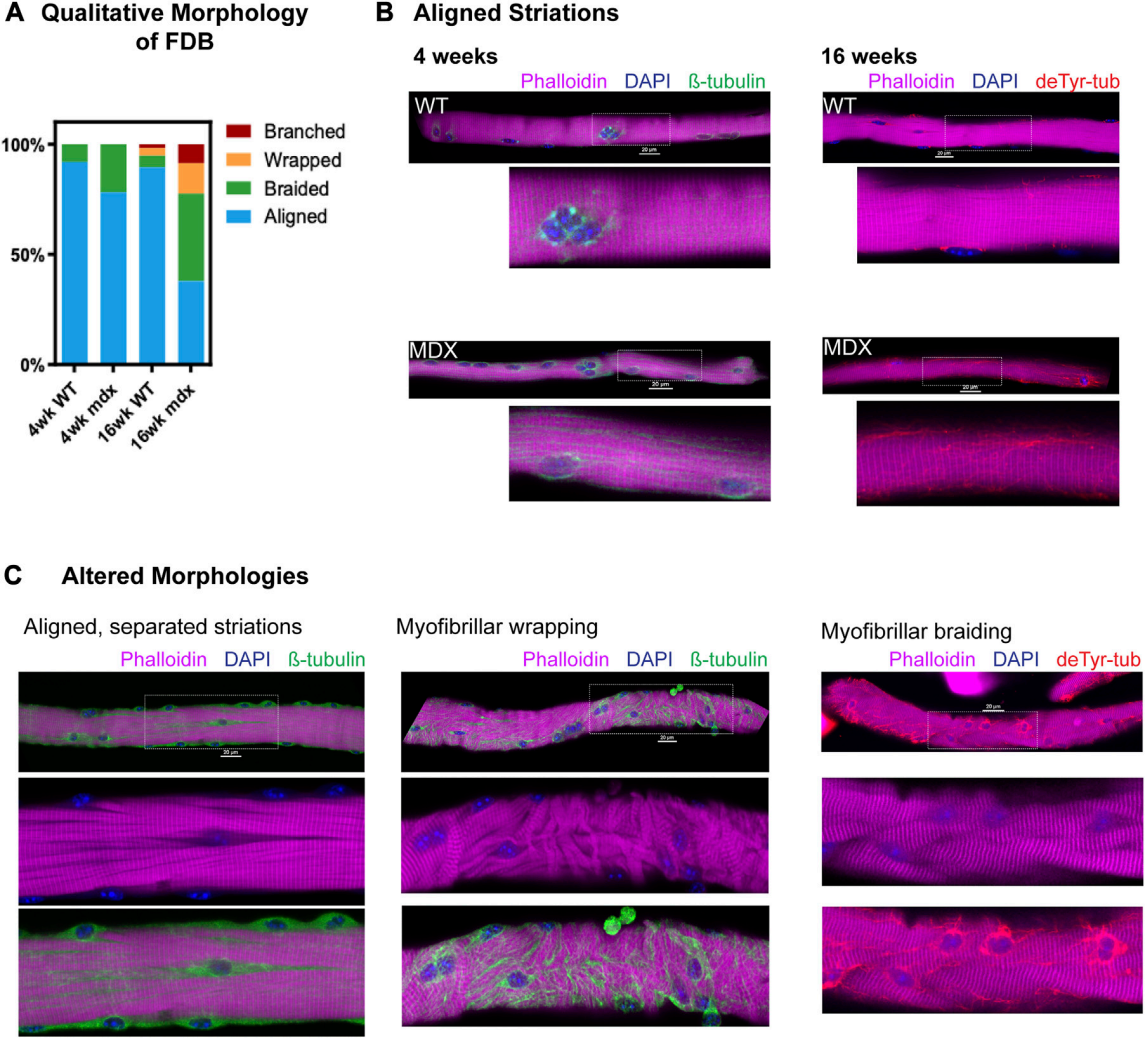
Qualitative survey of fiber morphology **(A)** The percent distribution of each morphology was determined based on a survey of approx. of 150 fibers (*n* = 5 mice/genotype/age, ∼30 fibers/animal) from each condition. **(B)** Representative images showing aligned striations at 4 and 16 weeks with canonical rectilinear MT structure. MT bundling is apparent in *mdx* as early as 4 weeks. Detyrosinated tubulin appears increased in 16 weeks *mdx*. **(C)** Fibers from 16 weeks *mdx* representative of altered morphologies, showing areas of MT bundling appearing coincident with alterations in striation continuity, in fibers with myofibrillar malformation as well as in fibers with otherwise aligned striations.

Alterations in myofibrillar structure are a consequence of deficient myofibrillar repair following acute muscle damage (McHugh and Tyler, 2019) or disease pathology (Head et al., 1997; Lovering et al., 2011; Buttgereit et al., 2013) that predisposes the occurrence of gross malformations in muscle structure (i.e., split and branched fibers) (Head et al., 1997). Our group previously reported a low percentage of grossly malformed (i.e., bifurcated, split, etc.) skeletal muscle fibers in 6–9 weeks *mdx*, with less than 10% abnormal fibers found in the flexor digitorum brevis (FDB) (Lovering et al., 2009; Goodall et al., 2012). These observations were made by visual inspection in brightfield where we found > 90% of the FDB muscle fibers had no detectable abnormalities. In the current study, we show that when labeled for myofibrillar structure (i.e., phalloidin labeled actin), and imaged with confocal microscopy, a more significant number of muscle fibers with abnormalities in myofibrillar structure becomes apparent (Figure 3).

Using an automated confocal strategy, we imaged single isolated FDB myofibers from WT and *mdx* at 4 and 16 weeks. Our qualitative visual inspection identified four distinct morphologies of myofibrils within the muscle fiber: 1) canonical aligned striations; 2) evidence of “braided” myofibril structure with misregistration; 3) misalignment characterized by myofibrils wrapping around the peripheral myofibrils; 4) fibers exhibiting gross malformations (i.e., branches, splits); as previously described (Goodall et al., 2012) (Figures 3A–C). In the 4 weeks *mdx*, myofibers with evidence of braided myofibrils make up 22% of the total FDB population. By 16 weeks, only 38% of myofibers in the *mdx* FDB exhibit canonical aligned striations; 39% of fibers have braided myofibrils, 14% have myofibrillar wrapping, and 9% of muscle fibers are branched (Figure 3C). Although muscle fibers with altered myofibrillar morphologies were identified in the wild-type, these comprised less than 11% of the total FDB population.

While fibers with canonical aligned striations were in the majority in both genotypes, a significant number of otherwise normal *mdx* fibers presented with separations between myofibrils, marked by bundles of microtubules (Figure 3C). In fact, these myofibrillar separations were evident in a majority of 16 weeks *mdx* muscle fibers and were observed in some wild-type FDBs albeit at a markedly reduced occurrence. These initial qualitative observations provided the basis for our adopting quantitative methods. Informed by our past observations of the MT densification often occurring in discrete areas in *mdx* muscle fibers (Khairallah et al., 2012 [Fig 2]; Kerr et al., 2015 [Fig 1]), and new qualitative evidence that this MT densification is coincident with altered myofibrillar structure, we posited a link between the disease altered MTs and the occurrence of myofibrillar malformations in DMD.

### 3.3 The continuity of Z-line striations as a metric of myofibrillar structure

We next quantified the continuity of myofibrillar Z-line striations as a metric of myofibrillar structure. The quantitative assessment of Z-line striations in each image was performed using a custom MATLAB routine established by Morris. (2021) for profiling myofibrillar structure in developing cardiomyocytes and skeletal myotubes and adapted here for mature skeletal muscle fibers.

**FIGURE 4:**
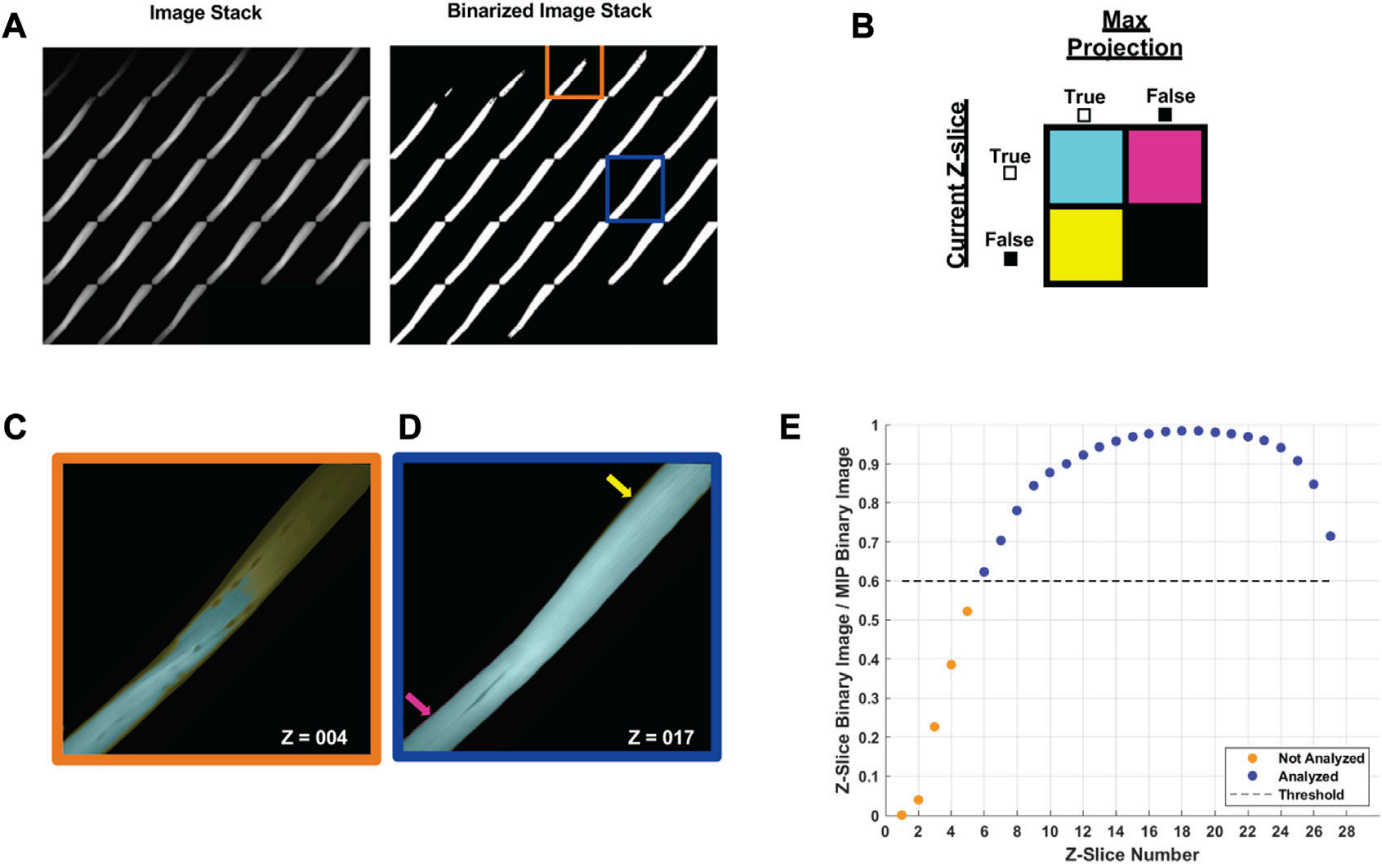
Automated image selection for myofiber analysis **(A)** Each z-slice is binarized and compared with the binarized maximum intensity projection (max-IP). **(B)** Within the current z-slice pixels with sufficient intensity for analysis are logged as true. The total number of true pixels in the current z-slice is compared as a ratio between true pixels in the max-IP. **(C)** A representative selection shows the fiber area of the fourth z-slice (outlined in orange) was less than 60% of the max-IP area whereas **(D)** the fiber area of 17th z-slice exceeded the 60% threshold. **(E)** A representative plot of the z-slices to be included in analysis based on the user defined threshold (nthresh = 0.6).

In brief, multi-channel Nikon confocal fluorescence images were converted into RGB TIFFs, and the Cy5 channel containing phalloidin-633 for actin was output to a new image stack. To decrease processing time, each image was cropped to the fiber of interest and rotated to align the myofiber long-axis to the *x*-axis of the frame. The maximal myofiber boundary was identified with an Otsu’s threshold (Otsu, 1979) of the maximum intensity projection (max-IP) of the z-stack (Figure 4A). In the resulting binary image, the myofiber area was calculated and the orientation axis was determined using the least mean square orientation estimation algorithm. Subsequently, each z-slice was binarized based on Otsu’s threshold and compared to the binarized maximum intensity projection to determine the ratio of “true” pixels in the z-slice of interest compared to the max-IP (Figures 4B–D). Z-slice ratios above a user-defined threshold (nthresh = 0.6) were selected for analysis (Figure 4E).

**FIGURE 5:**
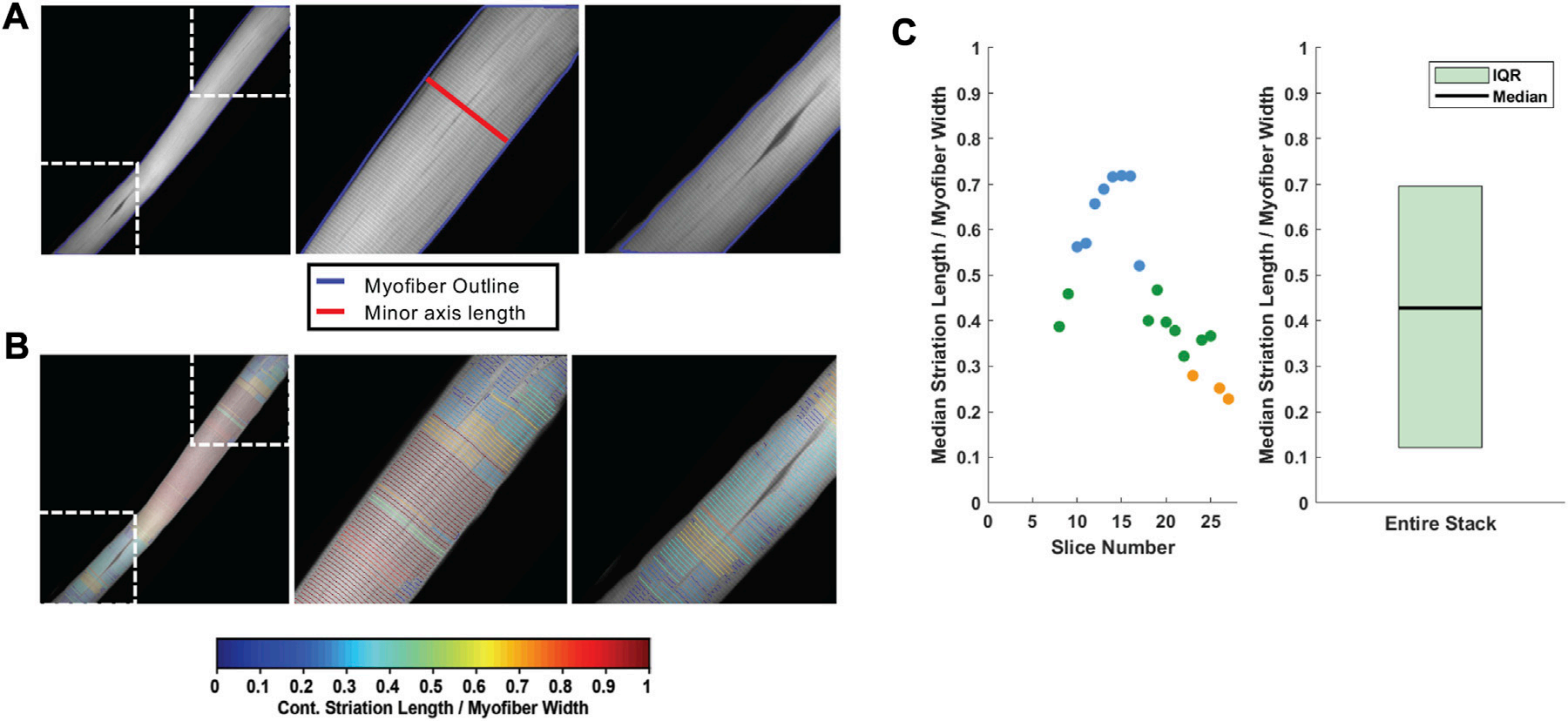
Striation continuity quantification. **(A)** Representative z-slice of phalloidin channel of WT FDB shown in Figure 4. Insets (right) showing Z-line structure. **(B)** Automated Z-line detection output from ZlineDetection. Insets (right) showing the heterogeneity of Z-line continuity in WT FDB at minor separations between myofibrils and the nucleus. **(C)** Quantification of the striation length as compared to myofiber width within each z-slice (left) and the corresponding box plot for the entire z-stack (right).

For each analyzed z-stack image, the minor axis length (i.e., width) of the myofiber was determined for every 20 pixels along the long axis of the muscle fiber (Figure 5A). Subsequently, the phalloidin labeled Z-line structure was detected via the ZlineDetection algorithm developed by Morris et al. (Morris et al., 2020). The continuity of each Z-line was determined by its length divided by the nearest minor axis length and plotted with color code (Figure 5B) with a continuous Z-line spanning the muscle fiber perpendicular axis yielding a measure of 1 (red), with interruptions in the continuity yielding lower values and cooler colors. The output for each fiber includes a mean striation continuity score for each z-slice as well as a boxplot for the entire z-stack (Figure 5C). The median striation length for the entire muscle fiber z-stack is also reported as the continuity score.

### 3.4 Z-line continuity decreases with dystrophic progression and predicts altered myofibrillar structure

**FIGURE 6:**
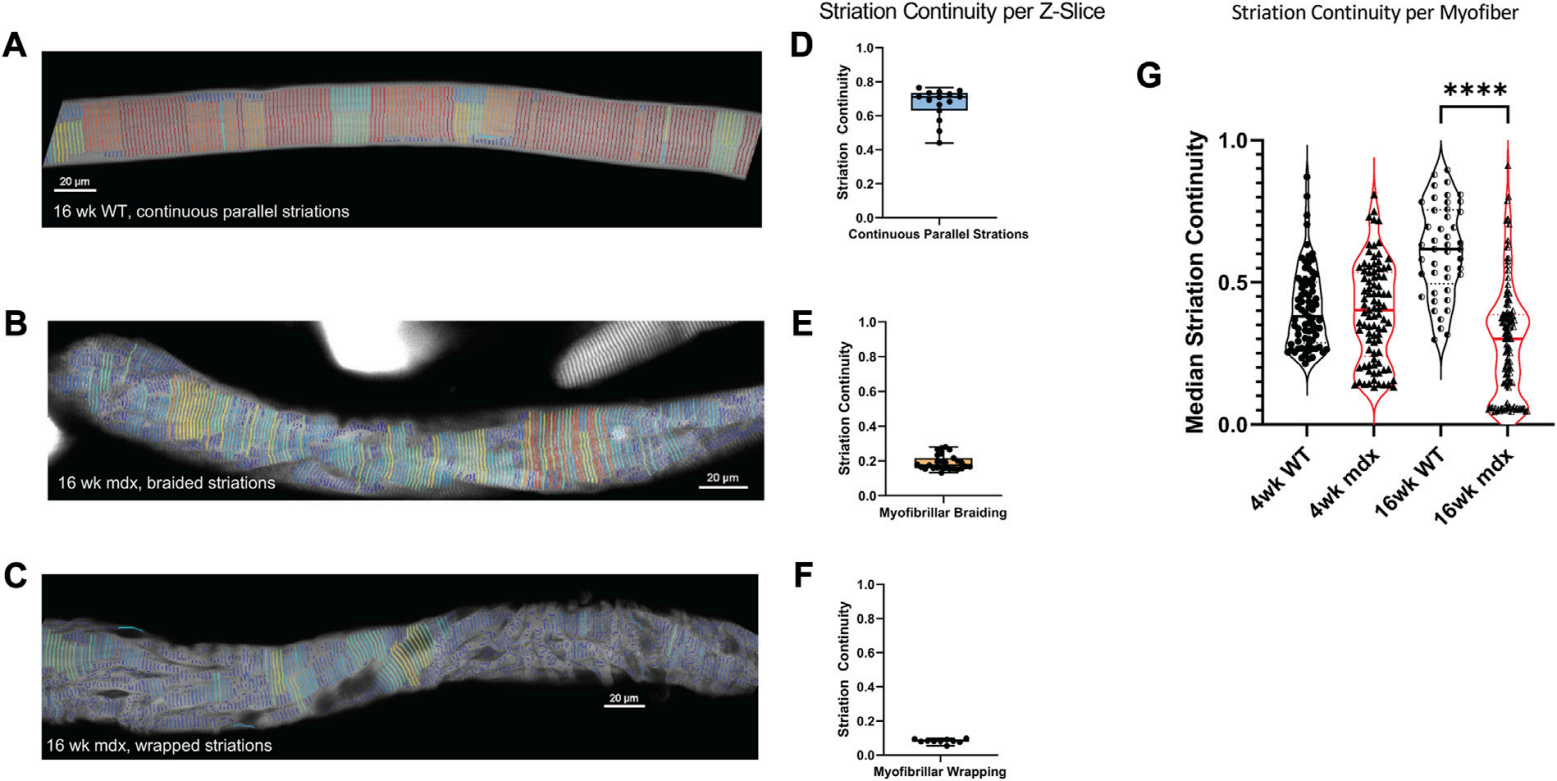
Median Striation Continuity scores decrease with altered morphology. Striation detection for **(A)** the full 16weeks wild-type fiber depicted in Figure 3, **(B)** the 16 weeks mdx fiber with braided myofibrils, and **(C)** the 16weeks mdx fiber with wrapped myofibrils. Their respective continuity scores per z-slice are illustrated with min, max, median scores displayed in boxplots **(D–F)**. **(G)** Average striation continuity scores for each fiber within each experimental group (*n* = 50–125 fibers per condition, across 5 mice). One-way ANOVA revealed significant difference between groups with Šídák’s multiple comparisons test elucidating a significant difference between 16 weeks WT and MDX (5 mice per age/genotype) (*F*(3, 333) = 38.36, *p* < 0.0001).

We show that striation continuity is a measure that effectively identifies the minor interruptions in the myofibrillar structure seen in WT muscle fibers as well as the more significant disruptions in the *mdx* (Figures 6A–F). We demonstrate continuity scores < 0.4 in fibers displaying areas of myofibrillar braiding (Figures 6B, D), while scores decrease to < 0.2 in fibers with significant myofibrillar wrapping (Figures 6C, F).

Using the automated imaging strategy and ZlineDetection to quantitate Z-line continuity, we screened FDB myofibers from WT and *mdx* mice at 4 and 16 weeks of age. At 4 weeks we find no significant difference in the mean striation continuity of the entire fiber between *mdx* and WT (Figure 6G), the 4-week *mdx* do achieve lower minimum continuity scores than the 4-week wild-type. Because severely misaligned myofibrils are a rare event at 4 weeks, the reduced continuity score is manifest from microtubule bundles between myofibrils causing separations as previously described (Figure 3A).

By 16 weeks, the differences between morphology in *mdx* and wild-type become more pronounced. While we found an increase in the average continuity score in wild-type, the continuity of the *mdx* decreased as altered morphologies emerge at a greater frequency (Figure 6G). This increase in separations between myofibrils in the *mdx* between 4 and 16 weeks suggests a progression of myofibrillar alterations with disease progression.

### 3.5 Increased tubulin detyrosination occurs commensurate with myofibrillar malformations

**FIGURE 7:**
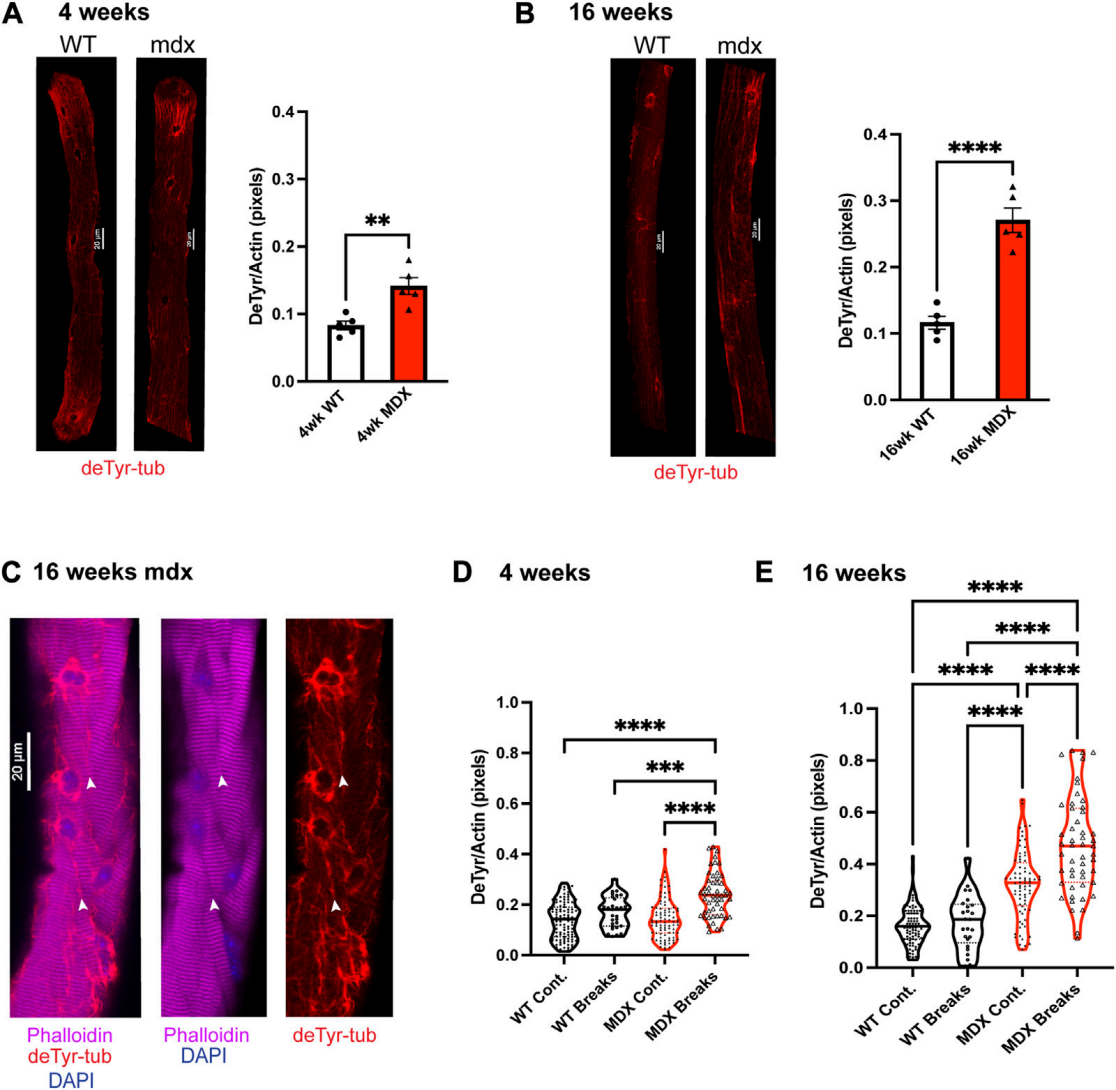
Detyrosination is enriched in dystrophic fibers and at the site of myofibrillar malformation. **(A,B)** We show the average deTyr density normalized to fiber size via the actin stain in fibers from wild-type and *mdx* mice 16 weeks (*n* = 5 mice). Values are means ± SEM. Statistical significance was determined using t-tests. **(C)** Representative images showing co-localization of detyrosinated tubulin with myofibrillar break sites. **(D–E)** Quantification of deTyr-tubulin density per myofiber in regions with continuous striations across myofibrils vs. within break sites. Analysis was completed using one-way ANOVA with Šídák’s multiple comparisons test (**p < 0.01; ***p <0.001; ****p <0.0001).

Examining FDB myofibers labeled for deTyr-tubulin and actin we find a significant increase in the density of deTyr-MTs in the *mdx* at 4 weeks that progresses at 16 weeks (Figures 7A, B). Revisiting our previous observation of bundled microtubules coinciding with myofibrillar separations, we quantified the density of deTyr-tubulin within the regions of myofibrillar separation versus in areas with otherwise normal myofibrillar connectivity (Figures 7C–E). At 4 weeks we find no difference in the density of deTyr -MTs between these areas in WT, but in the *mdx,* we find a significant increase in deTyr-MTs only in the regions of myofibrillar separation.

In 16 weeks WT muscle fibers we again find no difference in the density of deTyr -MTs within the regions of myofibrillar separation versus areas with otherwise normal myofibrillar connectivity. In the 16 weeks *mdx* we again find a significant elevation in deTyr-MTs in areas of myofibrillar separation but now find these changes in areas of otherwise normal myofibrillar connectivity as well. (Figure 7E). Taken together, these results suggest that deTyr-MTs become abundant first between myofibrils then progress more globally throughout the myofibrillar structure as disease progresses.

### 3.6 Overexpression of VASH2-GFP + SVBP models the increased tubulin detyrosination, cytoskeletal stiffness, and myofibrillar malformations established as pathognomonic in *mdx*

**FIGURE 8:**
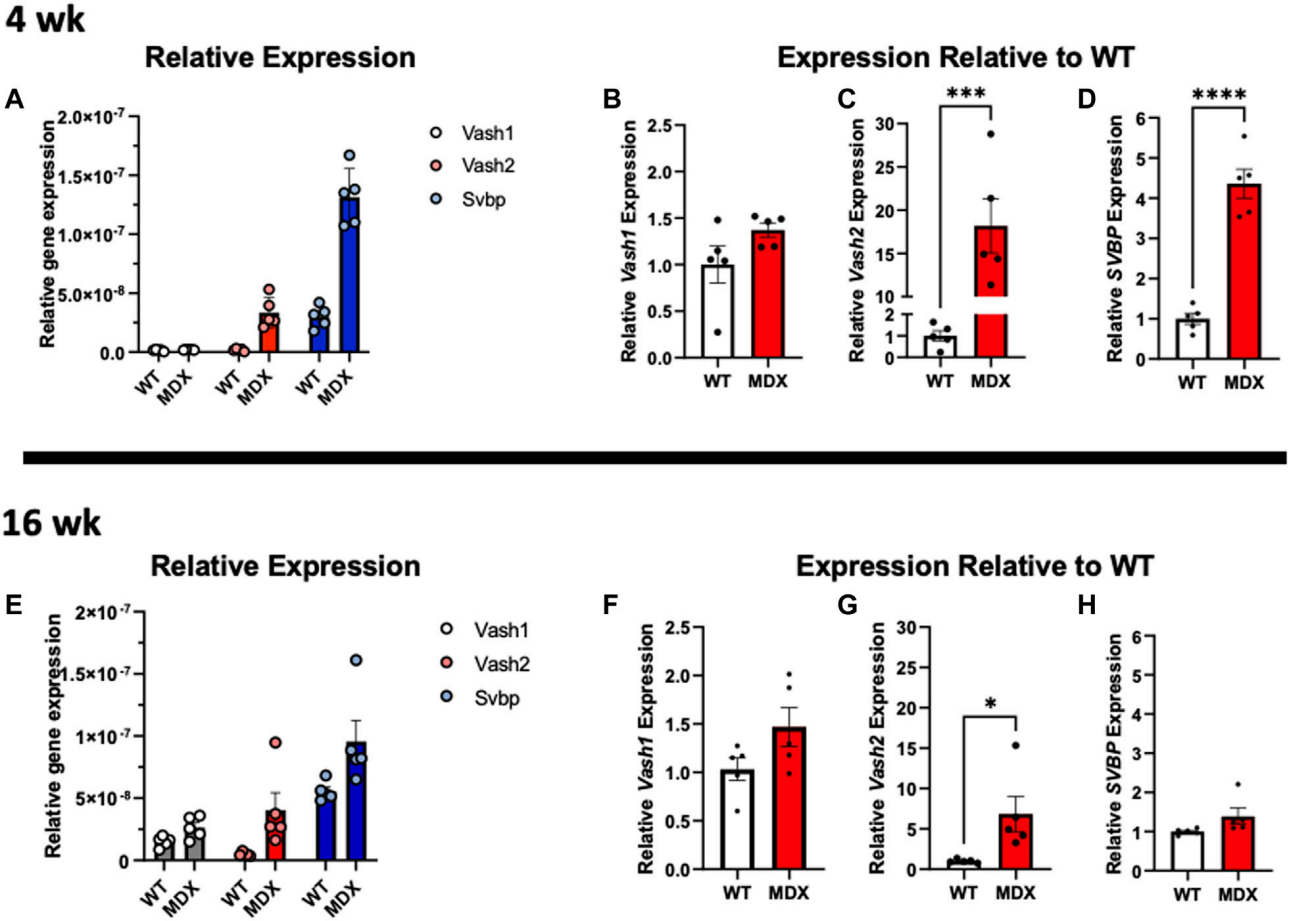
Expression of vasohibins and SVBP are increased in dystrophy. **(A–D)** Relative expression of VASH1, VASH2, and SVBP at 4 weeks reveals **(B)** VASH1 expression is not significantly increased at 4 weeks whereas **(C)** VASH2 expression is increased approximately 18-fold on average in the *mdx* and **(D)** and SVBP is increased approximately 4-fold. **(E)** Relative gene expression of both tubulin carboxypeptidases and especially VASH1, appears increased in wild-type at 16 weeks compared to 4 weeks and *mdx* expression relative to wild-type remains increased at 16 weeks **(F)** VASH1 shows a trend toward increased expression in *mdx*, only **(G)** VASH2 expression remains significantly increased, while its binding partner **(H)** SVBP is not significantly increased in *mdx* at 16 weeks. All analysis was performed using one-way ANOVA with Šídák’s multiple comparisons test (*p < 0.05; ***p <0.001; ****p <0.0001).

Detyrosination is the reversible enzymatic cleavage of the COOH-terminal tyrosine from α-tubulin by vasohibin 1 (VASH1) or vasohibin 2 (VASH2) and their partner the small vasohibin binding protein (SVBP) (Aillaud et al., 2017; Ramirez-Rios et al., 2022). Examining the transcripts of these proteins at 4 weeks (Figures 8A–D) finds no significant change in VASH1 yet a significant increase in VASH2 and SVBP in the *mdx*. At 16 weeks the VASH1 remained unchanged and VASH2 remained elevated in the *mdx.* In contrast was SVBP that was not different between genotypes at 16 weeks (Figures 8F–H).

**FIGURE 9:**
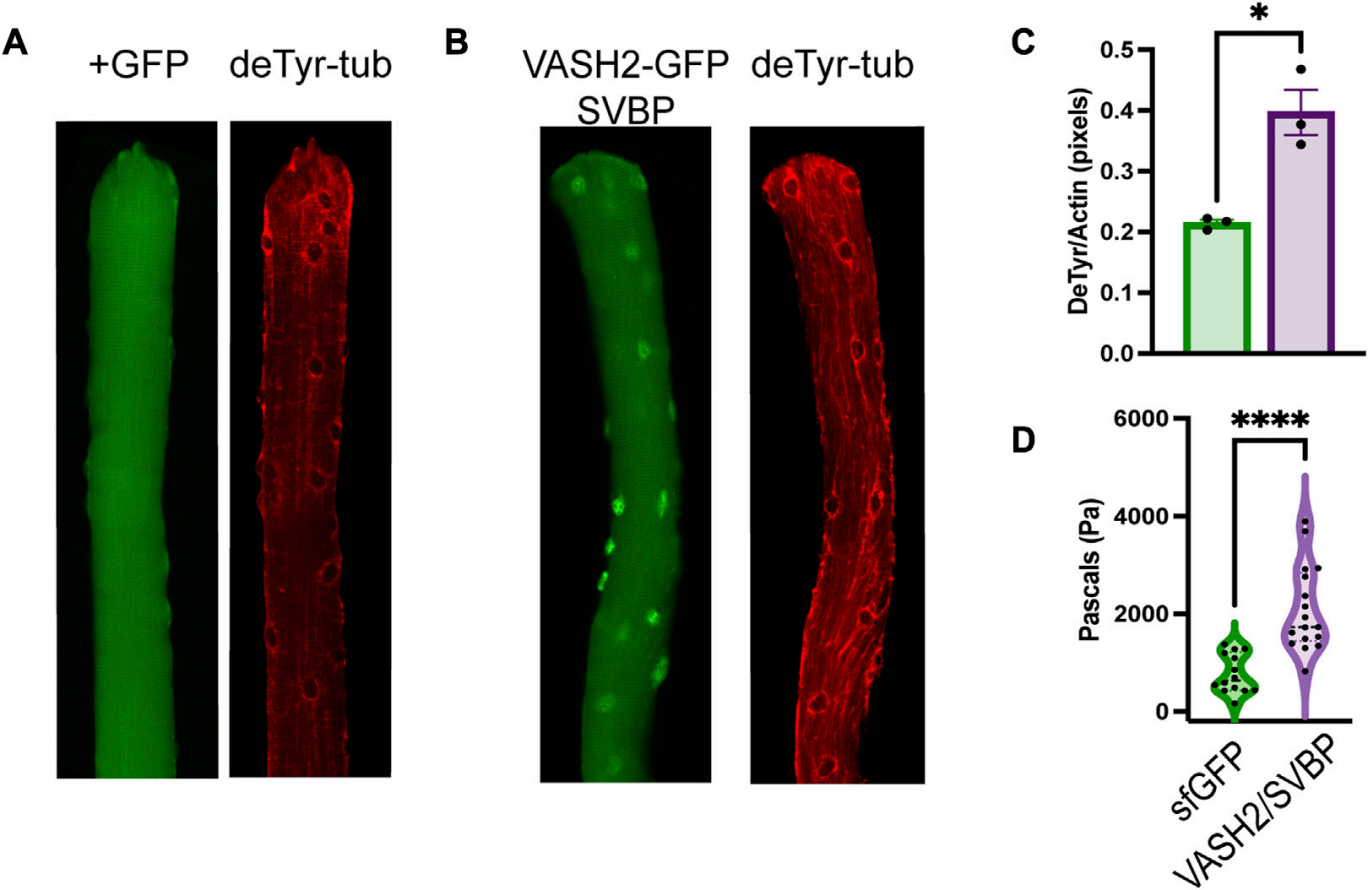
VASH2 overexpression increases the density of deTyr-MTs and the passive stiffness of the muscle fiber **(A)** Representative image muscle fiber expressing sfGFP(+) as a control finds deTyr-tubulin enriched MTs dispersed throughout the fiber. **(B)** VASH2+GFP expressing muscle fiber exhibits a denser and more bundled network of deTyr-tubulin enriched MTs. **(C)** Quantification of the density of deTyr-MTs finds a significant elevation with VASH2 overexpression (*n* = 3 mice per condition; 10–15 fibers per mouse). **(D)** Nano-indentation (5 μm/s) measure of viscoelastic resistance finds increased stiffness in VASH2-GFP muscle fibers. (*t*-test, *p < 0.05; ****p < 0.0001).

Microtubules are essential for myofibrillar growth, maintenance, and repair (Pizon et al., 2005; Scholz et al., 2008; Denes et al., 2021; Dhanyasi et al., 2021). We identified bundles of deTyr-modified MTs associated with myofibrillar separations in 4 weeks *mdx* muscle fibers that then progress to more significant changes at 16 weeks. Given this association we sought to determine if an experimental increase in deTyr-MTs in 4 weeks WT fibers was sufficient to recapitulate the changes seen in the 16 weeks *mdx*. To this end we used electroporation to introduce our bicistronic cDNA construct of VASH2-GFP + SVBP (Aillaud et al., 2017), or sfGFP cDNA as a control, into the 4 weeks old mouse FDB and examined the muscle fiber properties 5–7 days later (Figures 9A–C).

**FIGURE 10:**
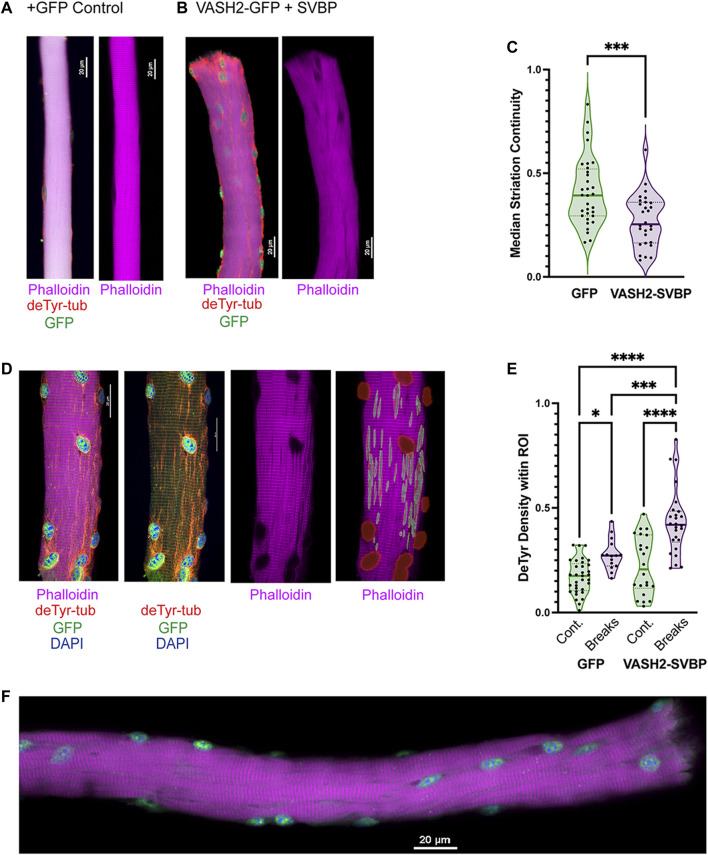
Detyrosinated microtubules are enriched at areas of Z-line disruption **(A,B)** Representative images of sfGFP and VASH-SVBP electroporated fibers respectively, with insets showing the disparate morphology. A closer look at the inset **(B)** reveals extensive veneering of striations, perhaps indicative of the onset of myofibrillar “braiding.” **(C)** Quantification of striation continuity using Z-line detection. There was a statistically significant difference between groups as determined by *t*-test **(D)** Representative image of sfGFP(+)VASH2 myofiber showing myofibrillar break sites identified as regions-of-interest (ROI) within NIS-Elements. **(E)** The density of the deTyr-MTs within each ROI was calculated and normalized to the respective area. There was a statistically significant difference between groups as determined by one-way ANOVA with Šídák’s multiple comparisons test (*F*(3,92) = 25.12). **(F)** Representative image of VASH2-SVBP electroporated FDB fiber with evidence of altered myofibrillar directionality (i.e., braiding). (*p < 0.05; ***p < 0.001; ****p < 0.0001).

In WT FDB fibers transduced with VASH2-GFP + SVBP, we find sfGFP localization in cytosol at both the m-line and with MT arrays (Figure 10D). We also find VASH2-GFP localized within the nucleus (Figure 9B; Figure 10D), a finding consistent with detyrosination as a regulator of mitotic spindle function (Barisic et al., 2015; Liao et al., 2019). Importantly, in WT FDB fibers transduced with VASH2-GFP + SVBP we find an increased abundance of deTyr-enriched MT arrays when compared to the controls expressing sfGFP controls (Figures 9A–C). In *mdx* muscle fibers we previously linked the elevated levels of deTyr-MTs to an increase in cytoskeletal mechanics (i.e., stiffness) (Kerr et al., 2015; Coleman et al., 2021). Using nanoindentation to measure the viscoelastic properties we show a significant increase in passive stiffness in VASH2-GFP + SVBP over expressing FDB muscle fibers compared to the sfGFP control (Figure 9D).

Our evidence suggested a link between the increased abundance of deTyr-enriched MT arrays and the altered myofibrillar structure in the 16 weeks *mdx*. We observed an increase incidence of sarcomere disruption in WT fibers overexpressing VASH2-GFP + SVBP compared to sfGFP expressing controls (Figures 10A, B). Consistent with this finding was a significant reduction in striation continuity in WT fibers overexpressing VASH2-GFP + SVBP determined by Z-line detection (Figure 10C). Concordant with our finding deTyr-enriched MTs co-registered with myofibrillar malformations in the *mdx* (Figure 7), our automated NIS-Elements analysis (Figure 10D) showed that VASH2-GFP + SVBP over-expression in WT myofibers yielded deTyr-tub enriched MT arrays only in areas with altered myofibrillar structure (Figure 10E). Further evidence supporting a link between deTyr-MTs and myofibrillar malformations came from visual inspection that revealed braided myofibrils in 21.3% of the VASH2-GFP + SVBP over-expressing myofibers (Figure 10F) with no evidence for these malformations in the GFP- controls. Given that VASH2-GFP + SVBP overexpression in WT muscle was sufficient to model the altered myofibrillar structure that arises in the 16 weeks *mdx*, we posit that the increased abundance of deTyr-enriched MT arrays is an early event in dystrophic pathology that predisposes the altered myofibrillar structure in dystrophinopathies.

## 4 Discussion

Altered myofibrillar structure is a consequence of dystrophic pathology in humans (Olivé et al., 2004; Murach et al., 2017) and rodents (Lovering et al., 2011; Buttgereit et al., 2013; Liu et al., 2013; Kiriaev et al., 2021) that impairs force production, decreases contraction velocity (Stefanati et al., 2021) and increases susceptibility to contraction injury. Informed by numerous experimental studies, optical predictions (Schneidereit et al., 2018) and mathematical models (Stefanati et al., 2021), myofibril misalignment and increased myofibrillar stiffness are thought the mechanisms that underscore these functional deficits. With the consequences of altered myofibrillar structure well defined, we sought to identify mechanisms that underlie their occurrence.

Here our focus was on microtubules (MTs) whose structure and properties are altered early in dystrophic disease (Prins et al., 2009; Khairallah et al., 2012; Belanto et al., 2014) and whose structure and properties play a critical role in myofibrillar growth, maintenance, and repair (Dhanyasi et al., 2021). Consistent with tubulin PTM’s as regulators of MT function, deTyr-enriched MT arrays have been implicated in the regulation of mechanotransduction-dependent ROS and Ca^2+^ signals (Kerr et al., 2015; Robison et al., 2016), in the directional transport of cargo (i.e., lysosomes) (Mohan et al., 2019), and in the highly orchestrated myofibrillar assembly during myogenesis (Gundersen et al., 1989; Chang et al., 2002). Here we show that myofibrillar malformations are not inherent to dystrophin’s absence, rather they arise in the *mdx* between 4 and 16 weeks of age coincident with the densification of deTyr-enriched MT arrays in these malformed areas. Transcriptional evidence of increased VASH2 and SVBP in the 4 weeks *mdx* suggested that the VASH2/SVBP complex may be may be indirectly responsible for the myofibrillar alterations by increasing the abundance of deTyr-enriched MT arrays. A causative link between deTyr-MT’s and myofibrillar alterations came from evidence showing VASH2-GFP + SVBP overexpression in WT muscle fibers sufficient to model the densification of deTyr-enriched MT arrays and altered myofibrillar structure in the 16 weeks *mdx*. Others have reported that both *mdx* and WT muscle reach terminal size by 14 weeks of age, with *mdx* exhibiting hypertrophy as well as progressive branching which is hypothesized to be preceded by myofibrillar malformations (Turk et al., 2005; Faber et al., 2014; Duddy et al., 2015; Massopust et al., 2020). Intriguingly, overexpression of deTyr-tubulin has also been reported commiserate with hypertrophy in striated muscle (Chen et al., 2018; Schuldt et al., 2021; Tian et al., 2022). While an exciting result, additional studies are needed to mechanistically explain how an increase in deTyr-enriched MT’s impacts myofibrillar structure.

This report extends our previous discovery implicating deTyr-enriched MT arrays as an early event in DMD pathology that drives the excess mechanotransduction elicited Nox2-ROS and Ca^2+^ signals linked to dystrophic progression (Khairallah et al., 2012; Prosser et al., 2013; Kerr et al., 2015). Together these results give strong support for disease-altered MTs as negative disease modifiers early in DMD pathology. Given the transcriptional and proteomic evidence for these alterations in muscle from DMD patients (Khairallah et al., 2012; Capitanio et al., 2020), targeted therapeutics to reduce deTyr-MTs may be a viable option to slow dystrophic progression. As pharmacologic approaches are developed, future studies to genetically reduce VASH1, VASH2, or SVBP in *mdx* fibers will further advance our mechanistic understanding.

While we focused on DMD in this study, it is notable that myofibrillar malformations were modeled in WT muscle fibers by VASH2/SVBP overexpression. This result demonstrates that dystrophin’s absence is not obligate in this process, nor is dysregulated signaling linked solely to dystrophic pathology. It is then tempting to speculate that the occurrence of myofibrillar malformations seen in aging muscle (Grounds, 2014; Pichavant and Pavlath, 2014), in disparate genetic diseases (Seto et al., 2011), and in conditions of supraphysiologic muscle growth (Antonio and Gonyea, 1994; Murach et al., 2019) may be driven by this same axis. In this regard, future work profiling these conditions, and mechanisms that increase VASH/SVBP expression and activity, will likely yield insights of broad importance.

## Data Availability

The original contributions presented in the study are included in the article/Supplementary Material, further inquiries can be directed to the corresponding author.
